# In Utero Development and Immunosurveillance of B Cell Acute Lymphoblastic Leukemia

**DOI:** 10.1007/s11864-022-00963-3

**Published:** 2022-03-16

**Authors:** Nadine Rüchel, Vera H. Jepsen, Daniel Hein, Ute Fischer, Arndt Borkhardt, Katharina L. Gössling

**Affiliations:** Department of Pediatric Oncology, Hematology and Clinical Immunology, Medical Faculty, Heinrich-Heine-University, Moorenstraße 5, 40225 Duesseldorf, Germany

**Keywords:** Acute lymphoblastic leukemia, Cancer predisposition, *ETV6-RUNX1*, High hyperdiploidy, Trained immunity, Preleukemic clone

## Abstract

Acute lymphoblastic leukemia (ALL) is the most frequent type of pediatric cancer with a peak incidence at 2–5 years of age. ALL frequently begins in utero with the emergence of clinically silent, preleukemic cells. Underlying leukemia-predisposing germline and acquired somatic mutations define distinct ALL subtypes that vary dramatically in treatment outcomes. In addition to genetic predisposition, a second hit, which usually occurs postnatally, is required for development of overt leukemia in most ALL subtypes. An untrained, dysregulated immune response, possibly due to an abnormal response to infection, may be an important co-factor triggering the onset of leukemia. Furthermore, the involvement of natural killer (NK) cells and T helper (Th) cells in controlling the preleukemic cells has been discussed. Identifying the cell of origin of the preleukemia-initiating event might give additional insights into potential options for prevention. Modulation of the immune system to achieve prolonged immunosurveillance of the preleukemic clone that eventually dies out in later years might present a future directive. Herein, we review the concepts of prenatal origin as well as potential preventive approaches to pediatric B cell precursor (BCP) ALL.

## Introduction

Leukemia is a life-threatening disease caused by uncontrolled proliferation of blood and blood precursor cells. Depending on the cell type of clonal expansion, it can be segregated into different subtypes that have quite distinct incidences, pathogenesis, treatment options, and outcomes [1]. Approximately one-third of all cancers diagnosed below the age of 18 are leukemia, with about 74% of these being acute lymphoblastic leukemia (ALL, 4.3/100,000 children <15 years) in Germany [2]. ALL peaks between the age of 2 and 5 years and has a good outcome in most cases. However, about 10% of children present with poor prognosis, based on subtype and risk factors like advanced age [3, 4]. Herein, we review and discuss recent studies and concepts of prenatal pathogenesis of leukemia, with a special focus on infections or microbiota influencing anti-leukemic immunosurveillance.

## Genetic susceptibility to childhood B cell precursor ALL (BCP-ALL)

Childhood B-ALL arises through a complex interplay between inherited genetic background and acquired somatic alterations [4]. The genetic background of patients includes alterations in cancer-predisposing genes, single nucleotide polymorphisms (SNPs), and cancer predisposition syndromes that confer susceptibility to leukemia [5]. In addition to the underlying inherited genetics, prenatal chromosomal aberrations, such as aneuploidy and interchromosomal translocations [6], give rise to preleukemic cells. Further oncogenic events in these clinically silent cell clones, most likely triggered by environmental factors in early childhood, are required to ultimately lead to overt leukemia [4].

## Leukemia-predisposing germline mutations

Several germline mutations which confer susceptibility to leukemia development have been described [7••]. Most of the affected genes are also targets of somatic alterations in ALL.

### Cancer-predisposing gene mutations

The transcription factor ETV6 is an important regulator of hematopoiesis [8]. Families with *ETV6* germline mutations often present with thrombocytopenia and susceptibility to hematologic malignancies, among which ALL is the most frequent [9, 10]. Most ALL cases with germline *ETV6* mutations belong to the hyperdiploid subtype [9]. *ETV6* germline mutations include missense, frameshift, nonsense mutations, deletions, and insertions, leading to a loss of function of ETV6 [5, 11]. A cluster of mutations occurs in the DNA-binding E26 transformation-specific (ETS) domain of *ETV6*, leading to dominant negative effects and transcriptional repression [5, 7, 12].

*PAX5*, located at 9p13, encodes for the B cell lineage transcription factor PAX5 which is important for B-lymphoid lineage maturation [13]. So far, only few families with germline *PAX5* mutations have been described, presenting with incomplete penetrance [14, 15]. Reported missense mutations of *PAX5* occur at amino acid positions G183 (c547G>A, p.Gly183Ser) or R38 (c113G>A, p.Arg38His), both resulting in decreased PAX5-mediated transcriptional repression [14–16]. Carriers of germline *PAX5* mutations are susceptible to acquiring ALL, but the presence of the mutation does not seem to be sufficient for development of overt leukemia. A second mutational hit is required, e.g., inactivation of the wild-type *PAX5* allele by deletion of 9p, formation of a 9q isochromosome, or dicentric 9q chromosome [14–16].

*IKZF1* encodes for the hematopoietic zinc-finger (ZF) transcription factor IKAROS. Germline *IKZF1* mutations have been described in families with common variable immunodeficiency (CVID) [17] and in cases of familial and sporadic ALL [18]. Mutations include missense, nonsense, and frameshift variants and are located mostly outside the ZF motifs [5]. *IKZF1* mutations within its DNA-binding domain affect transcriptional activation of its target genes, whereas truncating mutations may have an impact on dimerization [18]. The majority of identified *IKZF1* germline variants are not restricted to specific functional domains and were shown to impact subcellular localization, adhesion, and anti-leukemic drug efficacy [18].

### Cancer predisposition syndromes

Li-Fraumeni syndrome is an autosomal dominant disorder [19], usually caused by *TP53* germline mutations, that presents with high susceptibility to cancers like breast cancer, brain tumors, and ALL, predominantly low hypodiploid ALL [7, 20, 21]. Low hypodiploidy is characterized by 32–39 chromosomes and is present in approximately 1% of childhood ALL cases [7, 22]. Occurrence of germline *TP53* mutations is associated with older age at diagnosis and poor outcome [23]. *TP53* encodes the tumor suppressor protein p53 and is one of the most frequently mutated genes in cancer. The majority of *TP53* mutations occur in its DNA-binding or nuclear export domains [7, 20].

Children with Down syndrome or Noonan syndrome are also at higher risk of developing acute leukemia, primarily acute myeloid leukemia (AML) [24, 25]. Down syndrome is characterized by trisomy of chromosome 21, which may affect leukemia development [24]. About 1% of children with Down syndrome will develop ALL or AML [24]. Noonan syndrome is an autosomal dominant disorder that belongs to the family of RASopathies and presents with symptoms including facial dysmorphologies, growth retardation, heart defects, and skin manifestations [25]. Rarely, germline mutations in *PTPN11*, encoding the phosphatase SHP2, and in *SOS1*, encoding the guanine nucleotide exchange factor SOS1, have been observed in patients with Noonan syndrome, who subsequently developed ALL [25].

### Leukemia-predisposing SNPs

In addition to the rare but highly penetrant germline mutations and cancer predisposition syndromes described here, genome-wide association studies (GWASs) have identified further germline variations that are frequent but show low penetrance. These are mostly SNPs, which, cumulatively, may confer a higher risk for ALL development. Although these risk alleles individually produce a modest effect and may be of limited clinical significance, in aggregate they can give rise to as much as a ninefold increase in leukemia risk for subjects with risk alleles in multiple genes compared to subjects with no risk alleles [26]. Genes involved include *IKZF1*, *CDKN2A*, *PIP4K2A*, *LHPP*, *ELK3*, *GATA3*, *ARID5B*, *CEBPE*, *MYC*, *ERG*, and *TP63* [7, 27–30], with the SNPs being located in the vicinity of these genes and influencing gene expression. Some of these SNPs are associated with distinct ALL subtypes or genetic ancestry. Examples are an intronic SNP in *GATA3* (dbSNP: rs3824662) that is associated with Philadelphia chromosome (Ph)-like ALL and poor outcome [31] and a risk locus in *TP63* (dbSNP: rs17505102) that is associated with *ETV6-RUNX1*^+^ ALL [28].

## Prenatal somatic mutations in childhood BCP-ALL

Fusion genes generated by interchromosomal translocations are recurrent genetic alterations in pediatric BCP-ALL [32]. Several studies indicate that these translocations frequently arise in utero, giving rise to preleukemic cells. The first indications that ALL has prenatal origins were reports of concordant BCP-ALL in monozygotic twins [33–37]. In these cases, preleukemic cell clones arising in one twin spread to the other twin via the monochorionic placenta, as confirmed via the identification of shared genetic lesions, immunoglobulin (Ig), or T cell receptor (TCR) rearrangements in the leukemic cells of both twins [38]. Identification of genomic breakpoints in neonatal blood spots (Guthrie cards) or cord blood further corroborates the prenatal origin of preleukemic lesions [39–45]. Altogether, in utero development has been shown for several BCP-ALL subtypes, including high hyperdiploid ALL, *ETV6-RUNX1*, *BCR-ABL1*, *TCF3-PBX1*, and *KMT2A* rearrangements (as reviewed in [3•]).

### Hyperdiploidy

With up to 30% of cases, high hyperdiploidy is the most common genetic subtype in childhood BCP-ALL, characterized by the gain of chromosomes (>50 chromosomes) [22, 46]. While other tri- or tetrasomies have been reported, chromosomal gains typically include chromosomes X, 4, 6, 10, 14, 17, 18, and 21 [47]. The hyperdiploid genotype is likely generated by a single abnormal mitosis leading to simultaneous gain of chromosomes [48]. Leukemia susceptibility in high hyperdiploid ALL is driven by gene dosage effects [47, 49, 50] that impact chromatin architecture, e.g., by weakening topologically associating domain (TAD) boundaries [51•].

### ETV6-RUNX1

The most common chromosomal translocation of pediatric ALL, accounting for about 20% of cases, is t(12;21)(p13;q22) [52]. This translocation leads to the fusion of two transcription factors involved in normal hematopoiesis, *ETV6* and *RUNX1*. Although the *ETV6-RUNX1* translocation has been detected in a large number of healthy neonates (1-5%), leukemia incidence among carriers is much lower (0.2–1%) [3, 43]. The fusion gene has weak oncogenic potential that manifests itself in a low concordance rate of about 10% in monozygotic twins [38]. *ETV6-RUNX1* acts as an oncogenic transcription factor and leads to a specific preleukemic phenotype characterized by a partial block of B cell differentiation and aberrant co-expression of myeloid markers [53]. Recurrent postnatal, leukemia-inducing mutations include *ETV6* deletions (≈70% of cases), *RUNX1* gain (23%), and extra der(21)t(12;21) (10%) [54].

### BCR-ABL1

BCP-ALL with t(9;22)(q34;q11), also referred to as Ph^+^ ALL, is present in ≈2% of pediatric ALL, but is significantly more common in adults [22, 55]. The majority of pediatric patients with *BCR-ABL1* fusion genes harbor the p190 *BCR-ABL1* subtype [56]. This chromosomal translocation leads to the formation of the *BCR-ABL1* oncogene, encoding for a tyrosine kinase. While high hyperdiploidy and *ETV6-RUNX1* are associated with a favorable treatment outcome [57], *BCR-ABL1* confers a poorer outcome [58]. A common cooperating oncogenic lesion in *BCR-ABL1*^+^ ALL is the deletion of the B-lineage transcription factor *IKZF1* (in >80% of cases) [59].

### TCF3-PBX1

The t(1;19)(q23;p13) translocation encoding the *TCF3-PBX1* fusion gene is present in ≈4% of childhood ALL cases [55, 60]. *TCF3-PBX1*^+^ ALL is associated with a good prognosis but frequent central nervous system (CNS) relapse [61]. Like *ETV6-RUNX1*, the *TCF3-PBX1* fusion protein has low oncogenic potential and requires secondary, cooperating mutations for overt leukemia to develop [62].

### KMT2A rearrangements

*KMT2A* (or *MLL*: mixed-lineage leukemia) rearrangements of 11q23 with other chromosomes are typically found in infant BCP-ALL (children <1 year) [34, 63]. *KMT2A*-rearranged leukemia often present with CNS involvement and are associated with poor treatment outcome [63]. Fusion genes involving *KMT2A* are likely sufficient for leukemia development, as suggested by a high concordance rate in monozygotic twins [38] and rare detection of secondary, cooperative mutations [64].

## The preleukemic cell of origin in childhood BCP-ALL

Investigation of early BCP-ALL development is invaluable in identifying new targeted treatment options and approaches to preventing leukemic transformation. BCP-ALL originates in a single cell, with subsequent clonal expansion of premalignant cells that may acquire more malignant traits. Due to the covert early etiology of the disease and the complexity of prenatal leukemic development, identifying and characterizing the BCP-ALL cell of origin remains challenging. Several studies have tried to narrow down the cell in which the first preleukemia-initiating event preferentially occurs (Table 1). Although B cell blasts of different BCP-ALL subtypes often correspond to distinct developmental stages of normal B cell hematopoiesis, the first oncogenic event might occur at a different developmental stage. A subsequent differentiation arrest at a later cell stage or dedifferentiation of preleukemic cells place them downstream or upstream of their cell of origin. Dedifferentiation of preleukemic cells was for instance proposed for *TCF3-PBX1* translocations [65, 66].

**Table 1:** BCP-ALL preleukemia-initiating cells suggested by different studies (selection)

ALL subtype	Year	Study	Methods	Proposed preleukemia-initiating cell
Hyperdiploid	1997	Quijano et al. [67]	FISH detection of hyperdiploid cells in FACS-sorted cell populations	Stem cell (CD34^+^CD33^−^CD38^−^CD19^−^)
	1999	Kasprzyk et al. [68]	FISH detection of hyperdiploid cells in FACS-sorted cell populations	Lymphoid-committed progenitor cell
*ETV6-RUNX1*	2002	Hotfilder et al. [69]	FISH and RT-qPCR detection of *ETV6-RUNX1* in FACS-sorted cell populations, colony-forming assays	CD19^+^ lymphoid progenitor
	2004	Cox et al. [70]	Long-term in vitro culture and transplantation of FACS-sorted cell populations into mice	CD34^+^CD10^−^ or CD34^+^CD19^−^ cell
	2008	Hong et al. [71]	FISH detection of *ETV6-RUNX1* in FACS-sorted cell populations, transplantation of sorted cells into mice	CD34^+^CD38^−/low^CD19^+^ cell
	2014	Alpar et al. [72]	Sequencing of Ig/TCR loci in blast cells of monozygotic twins	Pro B cell or stem cell upstream of *RAG1/2*^+^ B-lineage cells
	2018	Böiers et al. [53]	In vitro differentiation and transcriptome analysis of an *ETV6-RUNX1*^+^ hiPSC model	CD19^-^IL7R^+^ fetal cell (lympho-myeloid potential)
*BCR-ABL1*	2005	Hotfilder et al. [73]	FISH and RT-qPCR detection of *BCR-ABL1* in FACS-sorted cell populations, colony-forming assays	lymphoid-committed stem cell (CD34^+^CD19^−^)
	2005	Castor et al. [74]	FISH detection of *BCR-ABL1* in FACS-sorted cell populations, transplantation of sorted cells into mice	Committed B cell progenitor (p190 *BCR-ABL1*)
	2017	Hovorkova et al. [75]	MRD analysis by PCR and detection of Ig/TCR rearrangements	Multipotent hematopoietic progenitor (in cases of CML-like disease)
*TCF3-PBX1*	2002	Wiemels et al. [65]	detection of breakpoint sequences (DNA from Guthrie cards), analysis of Ig/TCR loci	Pre B cell (potential postnatal origin)
	2008	Tsai et al. [76]	analysis of publicly available breakpoint sequences	Pro B/pre B cell
	2015	Fischer et al. [66]	FISH and RT-qPCR detection of *TCF3-PBX1* in FACS-sorted cell populations	Lymphoid-committed cell
*KMT2A*-r	2005	Hotfilder et al. [73]	FISH and RT-qPCR detection of *KMT2A-AFF1*^+^ in FACS-sorted cell populations, colony-forming assays	Lymphoid-committed stem cell (CD34^+^CD19^−^)
	2016	Barrett et al. [77]	Analysis of fetal cell populations of *KMT2A-AFF1*^+^ mice, colony-forming assays, repopulation assays	Fetal liver lymphoid-primed multipotent progenitor (LMPP)
	2019	O’Byrne et al. [78•]	single-cell transcriptomics, colony-forming assays	Fetal pre-pro B progenitor (CD10^−^)

*FACS* fluorescence-activated cell sorting, *FISH* fluorescence in situ hybridization, *Ig* immunoglobulin, *KMT2A*-*r KMT2A* rearrangements, *RT-qPCR* reverse transcription quantitative polymerase chain reaction, *TCR* T cell receptor, *RAG* recombination activating gene, *hiPSC* human induced pluripotent stem cell, *IL7R* interleukin-7 receptor, *MRD* minimal residual disease, *CML* chronic myeloid leukemia

An increasing number of studies provide evidence for the in utero origin of common BCP-ALL chromosome aberrations (as reviewed in [3•]). This suggests that preleukemic cells may arise in an early progenitor cell during fetal development, e.g., in the bone marrow or fetal liver.

Ig and TCR gene rearrangements in BCP-ALL blast cells have been used as markers to investigate the clonal origin of leukemic cells. These markers have been identified in a large number of BCP-ALL patients (>90%) [79, 80]. However, given that recombination activating gene (*RAG*)-driven rearrangements take place continually during clonal evolution of BCP-ALL [81], Ig/TCR gene status may not reflect the preleukemia-initiating cell. Shared clonal Ig and TCR gene rearrangements in twins with concordant BCP-ALL might give better insight, as shown in studies of twins with concordant *ETV6-RUNX1*^+^ ALL that identified pro B cells or *RAG1/2*^−^ stem cells as potential cells of origin [72, 82].

Lineage switching upon relapse has been described in BCP-ALL, mostly for *KMT2A*-rearranged or *BCR-ABL1*^+^ ALLs [83, 84]. In the latter case, a subgroup of patients carrying the fusion gene presented with chronic myeloid leukemia (CML)-like disease, pointing to a multipotent progenitor cell [75]. Likewise, ambiguous expression of lymphoid and myeloid lineage markers, as observed in many BCP-ALL patients [85], might point to an early progenitor cell with lympho-myeloid potential. Recently, lympho-myeloid precursor origin has been suggested for *ETV6-RUNX1*^+^ ALL, due to aberrant co-expression of myeloid markers observed in an *ETV6-RUNX1*^+^ human-induced pluripotent stem cell (hiPSC) model [53].

## Interleukin-7 receptor α (IL-7Rα) mutations in BCP-ALL development

IL-7Rα (encoded by the *IL7R* gene) is an important factor for lymphoid development. Together with the interleukin-2 receptor gamma (IL-2Rγ), it forms the IL-7 receptor (IL-7R) [86]. Recently, several groups have described activating mutations in *IL7R* as being involved in the initiation and development of BCP-ALL [87–89]. Inactivating mutations of *IL7R* are associated with severe combined immunodeficiency (SCID). SCID patients lack T cells. In mice, SCID manifests in both B and T cell absence [90]. In contrast, activating *IL7R* mutations have been observed in ALL, especially in Ph-like and *PAX5* P80R subtypes. Using a conditional knock-in mouse model, Almeida et al. showed that physiological levels of mutant IL-7Rα were sufficient to generate preleukemic B cell precursors and to initiate leukemia resembling the human Ph-like and *PAX5* P80R ALL subtypes [87]. Thomas et al. generated a genetically engineered mouse model with B cell-intrinsic expression of mutant *IL7R* that presented with development of BCP-ALL [88]. In an elegant study, Geron et al. transduced human CD34^+^ hematopoietic cells with mutant IL-7Rα. After transplantation into NOD/LtSz-*scid IL-2Rγ*^null^ mice, a preleukemic state with retained self-renewal capacity developed [89••]. In all three studies, additional mutations acquired during leukemia development were observed. These led to upregulation of IL-7R signaling (via the JAK/STAT5 or the PI3K/mTOR pathway), upregulation of oncogenes (e.g., *MYC, BCL2*), and downregulation of tumor suppressors (including *IKZF1*) [87–89]. Additionally, *CDKN2A* was silenced [89••], and recurrent somatic *KRAS* mutations which cooperate with mutant *IL7R* were observed [87, 88].

Taking all this together, a clear leukemia-initiating effect of constitutively active IL-7Rα could be observed in different mouse models as well as in human hematopoietic progenitors, with similarities to Ph-like and/or *PAX5* P80R BCP-ALL subtypes. However, further studies are needed to fully understand how the interplay with other mutations leads to the development of overt leukemia.

## External factors for the development of leukemia

For the development of overt leukemia, a multifactorial etiology is proposed where a combination of genetic susceptibility and external factors induces leukemic transformation. External factors such as radiation, smoking, and infections, amongst others, can play a role in utero or postnatally. Radiation and smoking have already been reviewed elsewhere [91, 92], associating high doses of ionizing radiation with ALL development and paternal smoking preconception and during pregnancy with an elevated risk for ALL.

### Infection

Infection has been suggested to be a likely trigger for ALL development. As postulated in the two-hit or delayed infection hypothesis by Mel Greaves [4], overt BCP-ALL requires an initiating mutation in utero (first hit) as well as a second postnatal mutation (second hit) [4]. In this model, the second hit is triggered by a dysregulated immune response towards common infections. Depending on the timing, infections were suggested to either have a protective (early) or detrimental (late) effect [4]. Pre- and postnatal infections have therefore been investigated as potential risk factors for triggering ALL.

In utero cytomegalovirus (CMV) infection was found to be more prevalent in children who later developed leukemia compared to healthy controls [93]. CMV is a member of the herpesvirus family and is known to cause hearing loss and/or growth retardation in the developing child [94, 95]. CMV can cross the placenta and thus infect the child in utero. Maternal reactivation or reinfection can also play a role, probably due to influences on immune crosstalk between mother and fetus [95]. Interestingly, CMV degrades the neonatal Fc receptor (FcRn) which is responsible for the transfer of IgG through the placenta. Thereby, CMV interferes with the immunity that is conferred from mother to child [96].

Other herpesviruses, like Epstein-Barr virus (EBV) and varicella zoster virus (VZV), may also play a role in the development of childhood BCP-ALL. A significantly increased risk for ALL development could be detected for maternal EBV infection [97]; however, significant correlation of EBV infection and ALL development could not be shown in a follow-up study [98]. A higher childhood leukemia risk was also observed when the mothers were infected with varicella or rubella during pregnancy [99•].

A link between maternal influenza infection and an increased risk of leukemia development was found in several studies as early as the 1970s [100, 101]. In a current meta-analysis by He et al., maternal influenza infection was significantly associated with higher risk of developing ALL [99•].

In terms of postnatal infections, a possible connection to influenza was observed in two space-time clusters [102, 103]. In the UK, increases in ALL incidence were observed in the years 1976 and 1990, following winter influenza epidemics [102]. In Milan, Italy, seven newly diagnosed ALL cases occurred within 4 weeks. All of these children were seropositive for the AH1N1 swine flu virus, whose outbreak occurred 3 to 6 months prior to leukemia diagnosis [103]. A possible explanation could be that influenza infection led to a strong dysregulated inflammatory response in the predisposed children, resulting in leukemic transformation of preleukemic cells. However, it is unlikely that influenza plays a unique role in the development of childhood ALL. It seems to be more important that predisposed children show an abnormal immune response to common infections. Other space-time clusters with a high incidence of childhood leukemia cases, e.g., the one in Fallon, USA (1997–2003), were not linked to influenza epidemics [104].

In light of the current severe acute respiratory syndrome coronavirus type 2 (SARS-CoV-2) pandemic, it will be interesting to see the influences of the virus and the infection-prevention measures (e.g., lockdown, increased hygiene) on the development of ALL cases in the future. The critical second hit for the development of overt leukemia could be infection with SARS-CoV-2, leading to an aberrant immune reaction [105]. However, it is also possible that the measures taken to prevent the spread of the disease, like closing nurseries and schools, may provide a means for reducing ALL cases. A similar scenario occurred during the SARS-CoV-1 outbreak in Hong Kong in 2003 [106]. Here, a marked decline in common infectious diseases, like chickenpox, as well as a decline in ALL incidence were observed in the same year [106]. However, the same measures could also lead to an increase of ALL cases in the next years, as children born during the current pandemic have fewer social contacts and are less exposed to common infections during the critical time period where the immune system has to be trained in order to avoid ALL development, according to the delayed infection hypothesis [4]. Thus, the next years will show the influence of the pandemic and of the lockdown measures on the development of ALL cases and may give initial insights into how to prevent the development of leukemia in the future.

Taken together, infections may promote leukemia at two different stages: (1) in utero due to the oncogenic potential of a virus or due to immune responses of a not yet fully developed fetal immune system or (2) after birth due to a dysregulated immune response.

## The in utero and early-in-life development of the immune system has long-term consequences for efficient control of the preleukemic clone

The double-hit scenario of secondary events, such as infections, triggering leukemic progression is supported by epidemiological data [4]. Additionally, animal studies showed that genetically predisposed mice developed leukemia only in a pathogen-containing environment [4, 107]. The exact mechanism remains unclear, but the lack of efficient immune cell training by microbial colonization and pathogens in utero and early in life has been suggested to be crucial for the development of ALL [5, 108].

Infections shape the immune system and thereby indirectly affect the preleukemic clone. In this context, among other innate immune cells, natural killer (NK) cells have been shown to be modulated by trained immunity. Infectious stimuli induced epigenetic reprogramming towards enhanced killing capacity of NK cells [109]. Furthermore, NK cells combine an antiviral and anti-tumor killing capacity and are thus promising candidates for modulation of preleukemic cells. NK cells were shown to gain memory functions after viral infections or after stimulation with pro-inflammatory cytokines [110, 111]. Interestingly, NK cell cytotoxicity against a leukemic cell line was also significantly enhanced after CMV infection, mediated by the NKG2C^(+)^ NK cell subpopulation [112]. In contrast, single cell RNA sequencing of *ETV6-RUNX1*^+^ ALL cases revealed significant inhibition of NK cell activity in the tumor microenvironment [113••]. This suggests that the dual role of NK cells can be explained by taking the different NK cell subtypes into account. Recent genetic studies have provided proof that a certain genetic constitution of NK cells controls BCP-ALL [114]. Killer immunoglobulin receptors (KIR) on NK cells interact with human lymphocyte antigen (HLA) class I molecules. The inhibitory NK cell receptor KIR2DL1—a high-affinity ligand for HLA-C2—is significantly increased in BCP-ALL patients. In another study, five NK cell-related factors (KIR2DL5A, NKp46, FasL, granzyme B and PI-9) were positively associated with detection of minimal residual disease at the end of induction therapy [115]. How the inhibitory NK cell receptors’ control of the preleukemic clone is determined by genetic factors or modulated by infections should be part of future studies.

**Figure 1:**
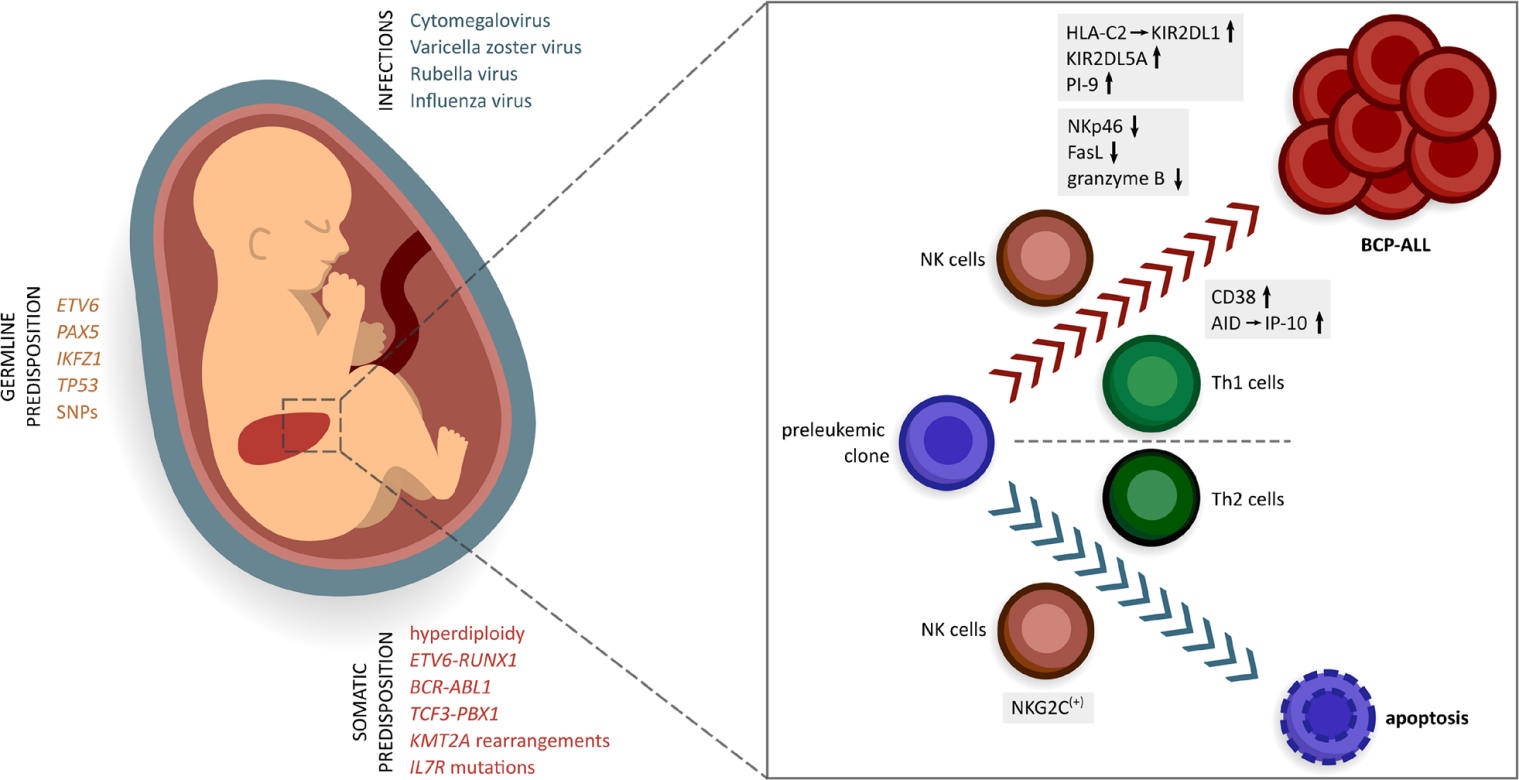
Immunosurveillance of the preleukemic clone**.** Germline and acquired somatic mutations predispose towards leukemia and define distinct ALL subtypes. Via a dysregulated immune response, infections can trigger transformation of the preleukemic clone into overt leukemia. This process is under constant immunosurveillance. T helper (Th) 1 cells can favor leukemia development via upregulation of CD38 and interferon gamma-induced protein 10 (IP-10), mediated by activation-induced cytidine deaminase (AID). Th2 cells on the other hand can inhibit leukemia development. Natural killer (NK) cells play an important role in cancer surveillance. They can favor development of overt leukemia by up- or downregulation of different factors, such as HLA-C2, KIR2DL1, KIR2DL5A, PI-9, NKp46, FasL, and granzyme B. Apoptosis of the preleukemic clone can be mediated by NKG2C^(+)^ NK cells. SNP, single nucleotide polymorphism; *IL7R*, interleukin-7 receptor alpha.

The importance of early and even prenatal immune training with microbial antigens is underlined by epidemiological data that refer to the hygiene hypothesis [116•]. Interestingly, the same epidemiological factors leading to a clean and hygienic environment, such as late introduction into day care, order and number of siblings, and early antibiotic treatment [117], have been associated with a higher incidence of autoimmune diseases and allergies as well as with a higher incidence of BCP-ALL [4, 118]. These are all diseases that are predominantly mediated by T helper (Th) cells, suggesting a certain role of Th responses in the control of the preleukemic clone. Atopic disease and childhood ALL are negatively correlated. A Th2 phenotype might be protective against ALL development [119], while pro-inflammatory Th1 cells with high interferon gamma (IFNγ) levels have been shown to migrate towards BCP-ALL cells and favor their proliferation via upregulation of CD38 and IFNγ-induced protein 10 (IP-10) production [120••] mediated by activation-induced cytidine deaminase (AID) upregulation [121]. But, what driving force skews the immune response towards one or the other direction, given the fact that early immune cell priming is lacking in both scenarios? Miedema and colleagues attributed this to a particular genetic predisposition, since they found two SNPs in the *TLR6* gene associated with BCP-ALL, leading to an altered Th1/Th2 balance upon microbial exposure [122]. The immunosurveillance mechanisms are summarized in Figure 1.

## Treatment options and outlook

Diagnosis of a severe underlying germline ALL predisposition with a high penetrance, such as *TP53* mutation/Li-Fraumeni syndrome, offers the opportunity to monitor the patient closely for early cancer occurrence and clearly improves overall survival [123]. By contrast, diagnosis of a more common predisposition, like an in utero occurring somatic *ETV6-RUNX1* mutation, does not provide such a benefit, as the mutation confers only a minor risk of a child developing ALL, a disease for which current chemotherapy treatment protocols achieve 80–90% overall survival without early detection being critical for its outcome. However, successful treatment comes at the price of significant acute and late toxicities, which account for a large proportion of deaths. Acute adverse effects during chemotherapy for childhood cancer can affect all organs, and two-thirds of childhood cancer survivors live with long-term effects of the toxic treatment, which can be severe (e.g., cognitive impairment, osteonecrosis, secondary cancers, infertility, depression) [124]. Therefore, there is an urgent need to employ strategies aimed at preventing children from getting cancer in the first place. In the absence of means to directly target and eliminate the preleukemic cells, general training of the immune system early in life (e.g., in child day-care, through contact with pets) is recommended and promising. More targeted approaches currently include (1) training of the innate immune response via specific vaccination or (2) modulation of the microbiome (by, e.g., probiotics) to achieve a healthier, more complex state [5]. Targeting Th1/Th2 lineage determination to prevent the clonal expansion of the preleukemic clone may be a promising alternative treatment approach to follow up on. However, differentiation programs are complex and intricately cross-linked. Side effects of pharmacologic modulation in genetically predisposed children can be severe and may outweigh potential benefits.

We believe that further studies employing larger cohorts of predisposed children are clearly necessary to understand the complex interplay of genetic predisposition and environmental factors and to finally enable us to develop targeted preventive approaches.
